# Correlations Between Clinical, Anthropometric and Nutritional Evaluations in Patients with Parkinson’s Disease from Ghana: A Cross-Sectional Study

**DOI:** 10.3390/jcm15072686

**Published:** 2026-04-02

**Authors:** Carlotta Bolliri, Luca Magistrelli, Francesca Del Sorbo, Anna Zecchinelli, Daniela Calandrella, Momodou Cham, Elikem Ame-Bruce, Emanuele Cereda, Chiara Pusani, Ioannis Ugo Isaias, Michela Barichella, Gianni Pezzoli

**Affiliations:** 1 Fondazione Pezzoli per la Malattia di Parkinson, 20125 Milan, Italy; bolliri@parkinson.it (C.B.); calandrella@parkinson.it (D.C.); emanuele.cereda@virgilio.it (E.C.); ioannis.isaias@asst-pini-cto.it (I.U.I.); michela.barichella@asst-pini-cto.it (M.B.); pezzoli@parkinson.it (G.P.); 2 Parkinson Institute Milan, ASST G.Pini-CTO, 20126 Milan, Italy; francesca.delsorbo@asst-pini-cto.it (F.D.S.); anna.zecchinelli@asst-pini-cto.it (A.Z.); chiara.pusani@asst-pini-cto.it (C.P.); 3 Richard Novati Catholic Hospital, Sogakope P.O. Box SK 95, Ghana; modoufa@gmail.com (M.C.); elikembruce@gmail.com (E.A.-B.); 4 Clinical Nutrition and Dietetics Unit, Fondazione IRCCS Policlinico San Matteo, 27100 Pavia, Italy

**Keywords:** Parkinson’s disease, sarcopenia, malnutrition

## Abstract

**Introduction:** Malnutrition and sarcopenia are commonly observed in African patients with Parkinson’s disease (PD); however, limited data exist regarding the nutritional status and body composition of PD patients in sub-Saharan Africa. This study aims to describe the clinical, nutritional, and anthropometric characteristics of PD patients from Sogakope, in the Volta Region of Ghana. **Methods:** A total of 20 PD patients were recruited. All participants underwent comprehensive clinical and nutritional assessments. Motor symptoms were evaluated with the Unified Parkinson’s Disease Rating Scale (UPDRS). Dealing with non-motor symptoms, constipation was diagnosed according to the Roma III Criteria while dysphagia was assessed using the Swallowing Disturbance Questionnaire. The presence and impact of sialorrhea was determined using the Sialorrhea Clinical Scale. Nutritional assessment was performed with the Mini Nutritional Assessment short form (MNA-sf). Body composition parameters were measured using Bioelectrical Impedance Analysis (BIA), and muscle strength was evaluated with the Handgrip Strength Test. Correlations were assessed by Pearson or Spearman correlation analysis, as appropriate. Partial correlation analysis controlling for significant clinical variables was also performed. **Results:** Daily caloric intake was significantly lower compared to Western populations and was associated with a reduced body mass index (BMI) and body fat percentage. Constipation (80%) and sarcopenia (45%) were highly prevalent, whereas dysphagia was reported in only 15% of participants. Over 75% of patients were at risk of malnutrition. A significant inverse correlation was found between thigh circumference and disease duration (*r* = −0.517; *p* = 0.02). Additionally, protein intake (g/kg/day) was inversely correlated with motor symptom severity, as measured by the UPDRS Part III in the ON state (*r* = −0.544; *p* = 0.02). **Conclusions**: This study demonstrates a high prevalence of nutritional deficiencies, sarcopenia, and altered body composition in Ghanaian PD patients. These nutritional impairments are significantly associated with disease duration and motor symptom severity. The findings highlight the urgent need for early nutritional screening and intervention as part of a multidisciplinary approach to Parkinson’s disease management in resource-limited settings.

## 1. Introduction

Parkinson’s disease (PD) is a common neurodegenerative disorder characterized by bradykinesia, rigidity, and resting tremor. The disease generally has a unilateral onset and gradually spreads to the contralateral part. Besides the classical motor symptoms, patients often present other non-motor symptoms (NMSs) that can even precede the onset of the motor phenotype and may determine a great impact on patient’s disability and on the caregiver’s burden [1]. Hyposmia, constipation, depression and REM sleep behaviour disorder are frequently also present in the pre-clinical phase of the disease and may delay the correct neurological diagnosis. Furthermore, NMSs play an important role in the so-called complicated phase: they can exacerbate the off phases and may require specific therapeutic approaches (like deep brain stimulation or subcutaneous infusion of levodopa). NMSs have gained importance in recent years; therefore, they have been included as important endpoints in clinical trials [2]. Moreover, patients may also present dysphagia and sialorrhea, thus impacting food intake with consequent weight loss and reduced muscular mass, which is more pronounced in the later stages [3].

PD is characterized by the loss of dopaminergic neurons in the pars compacta of the substantia nigra. Unfortunately, up to now only symptomatic therapies are available, with levodopa representing the gold standard.

Its global prevalence is projected to increase significantly in the coming years, with major implications for public health systems, economies, and society at large. This may also be related on the one hand to increased life expectancy and on the other to more accurate neurological evaluations.

Despite this global trend, the incidence and prevalence of PD appear to be lower in certain regions, particularly sub-Saharan Africa. Recently published data show a prevalence of PD in this area ranging from 7/100,000 in Ethiopia to 67/100,000 in Nigeria. Other countries present an intermediate prevalence, like the one reported in Togo (20/100,000) [4]. On the contrary, North African countries display a higher prevalence. For example, Safiri and colleagues reported a prevalence of about 82/100,000 in Middle East and North Africa region [5] with higher values in specific populations and countries [6].

These differences may be attributed to genetic and environmental differences, as well as limited access to healthcare and diagnostic services, particularly in the Sub-Saharan area [7].

We previously reported on the dietary habits and nutritional status of a cohort of PD patients from Ghana, observing a lower daily caloric intake and reduced anthropometric measures—such as body weight and body mass index (BMI)—compared to Western populations [8]. These findings, often compounded by low levels of physical activity, may predispose individuals to sarcopenia. Sarcopenia has recently been associated not only with worsening motor symptoms but also with cognitive decline and decreased quality of life in individuals with Parkinsonism [9].

Different studies have pointed out that sarcopenia and malnutrition are commonly detected in PD patients. Accordingly, Paul and colleagues analyzed a cohort of 75 PD patients and 35 age- and gender-matched controls and found that risk of malnutrition, evaluated with Mini-Nutritional Assessment (MNA) Scale, was higher in patients (45.3% vs. 14% in controls) and none were malnourished. Interestingly, malnutrition significantly correlated with gastrointestinal symptoms like constipation, sialorrhea and dysphagia [10].

Growing evidence shows that muscle loss, anthropometric decline, and malnutrition are also highly prevalent in other neurodegenerative conditions, including Alzheimer’s disease (AD) and amyotrophic lateral sclerosis (ALS). Particularly, recent findings highlight that patients with AD, confirmed by consistent cerebrospinal fluid biomarkers, were malnourished and presented lower Fat Mass compared to patients suffering from dementia due to other causes (like fronto-temporal vascular dementia, Lewy Body Disease, etc.) [11]. Furthermore, in a recently published systematic review, 33.97% of 5293 AD patients were at risk of malnutrition while 3.74% were malnourished, assessed with the Mini Nutritional Assessment [12]. Amyotrophic lateral sclerosis (ALS) is a rare condition characterized by a rapid degeneration of upper and lower motoneurons with a rapid progression. Malnutrition is frequently described in this disease. Several studies pointed out that ALS patients present a “hypermethabolic” state which leads to weight loss and sarcopenia and represents an important negative prognostic factor [13]. Another study identified an overall prevalence of malnutrition of about 20%, and this can even reach 50% in the later stages of the disease. Notwithstanding, an increase in death risk is significantly and inversely correlated with the weight loss [14].

These findings suggest that sarcopenia represents a multidimensional geriatric and neuropsychiatric vulnerability rather than a condition specific to PD.

Nutritional intake is also known to influence pharmacological treatment outcomes. In particular, high-protein diets can interfere with the absorption of levodopa, the cornerstone therapy in PD, potentially diminishing its motor benefits [15]. Additionally, recent research by Kittipongphakorn et al. has identified inverse correlations between lower-limb circumferences (e.g., calf and thigh) and both the Unified Parkinson’s Disease Rating Scale (UPDRS) Part II scores and quality-of-life metrics, suggesting that reductions in muscle mass in these regions may negatively affect motor function and daily activities [16].

In this study, we aim to investigate, for the first time, the associations between clinical, anthropometric, and nutritional parameters in a cohort of PD patients from Ghana.

## 2. Materials and Methods

The data here presented are part of a study conducted in Ghana at the Richard Novati Hospital in Sogakope, located in the Volta region, and performed according to the good clinical practice guidelines and the Declaration of Helsinki. Informed consent was obtained from all of the patients (local Ethical committee: protocol number CHAG-IRB07022024). Patients were included if they were diagnosed with an idiopathic PD according to the current diagnostic criteria [17] and were recruited by a Medical Doctor with expertise in the field of movement disorders. Moreover, exclusion criteria were represented by present or past history of neoplasms and/or already known metabolic disorders (apart from diabetes mellitus).

All patients were in OFF-drug state at the time of clinical (both neurological and nutritional) evaluations (as requested by the clinical protocol). Subsequently, neurological assessment was performed after the morning intake of levodopa. In few cases, clinical data were collected with local translators because of language barriers. The assessment of nutritional status was carried out using various anthropometric measurements. Body weight was measured using a calibrated scale under standard conditions, with the patient being without shoes, while height was recorded with the help of a stadiometer. These values were then used to calculate the Body Mass Index (BMI), applying the standard formula (BMI = kg/m^2^). BMI was categorized according to the World Health Organization (WHO) guidelines: underweight (BMI < 18.5), normal weight (18.5–24.9), overweight (25–29.9), and obesity (≥30). In addition to these measurements, body circumferences were also evaluated using a flexible tape measure. Waist circumference was measured at the midpoint between the iliac crest and the last rib, while hip circumference was taken at the widest point of the hips. Arm circumference was measured on the non-dominant arm, specifically at the midpoint between the acromion and the olecranon with the elbow bent at a 90-degree angle. Finally, calf circumference was measured at the thickest part of the calf.

Body composition data were obtained using bioelectric impedence (BIA; AKERN 101 Biavector^®^, Florence, Italy): a whole-body vector analysis system for semi-quantitative assessment of body composition based on the measurement of resistance and reactance. BIA was performed in supine position, fasting conditions, and after removing any metallic items. The following parameters were collected: resistance reactance and impedance; Phase Angle (PA) was calculated, and Fat-Free Mass (FFM), Fat Mass (FM), Total Body Water (TBW) and Appendicular Skeletal Muscle Mass Index (ASMI) were estimated.

Sarcopenia was defined according to the algorithm of the European Working Group on Sarcopenia in the older people (EWGSOP2) [18]. Since no specific criteria have been established so far for the African population, the currently available ones were applied for our patients. Briefly, the definition is based on three main issues: low muscle strength (in our study evaluated with handgrip strength using a handgrip dynamometer—defined pathologic if lower than 27 kg in men and 16 kg in women), low muscle quantity (evaluated with biometrical impedance analysis; considered pathological when lower than 7 for men and 5.5 for women) or quality and low physical performance (using the adapted 4 m walking test). Regarding muscle strength, three measurements were taken for each patient, and the mean value has been considered. Risk of sarcopenia is defined by a reduced ASMI with normal handgrip strength; probable sarcopenia is identified when handgrip strength is reduced but ASMI is within the normal range; and confirmed sarcopenia is diagnosed when both parameters (handgrip strength and ASMI) are reduced.

To identify the presence or the risk of malnutrition, a short form of the Mini nutritional Assessment (MNA SF) was administered. It is a validated nutrition screening and assessment tool that can identify, among geriatric patients, those malnourished or at risk of malnutrition [19,20,21,22,23].

According to the total score, patients can be classified as follows: 0–7: malnourished, 8–11 risk of malnutrition, 12–14 normal nutritional status.

To identify the presence of dysphagia, the Swallowing Disturbance Questionnaire (SDQ) [24], validated for PD, was used. The scale has 14 items, each with a score ranging from 0 to 3, except one question with a score ranging from 0.5 to 2.5. The total score ranges from 0.5 to 44.5. The scientific evidence has suggested that it can be used for the diagnosis of dysphagia needing treatment (cut-off score 11). Constipation was assessed according to the Rome III criteria (Constipation module) [25]: patients are considered to suffer from functional constipation if their score is at least 2 points.

The Bristol Stool Scale (also known as the Bristol Stool Form Scale) is a medical tool used to classify the appearance of human stools into seven types, based on their shape and consistency. It helps assess bowel function and can indicate conditions such as constipation, normal transit, or diarrhea. Accordingly, stool types 1–2 indicate constipation, types 3–4 indicate normal bowel function, while types 5–7 indicate loose stools or diarrhea [26].

Daily nutritional intake was evaluated using a food intake diary adapted to the local contest. Results were then analyzed using a Metadieta validated software version 4.7 [27].

A food-frequency questionnaire was also administered to assess the qualitative intake of nutrients and the quantitative intake of food expressed in kilocalories. The questionnaire was drafted by the nutritionist, after having established the composition of the main typical dishes and collecting information from the local population.

### Statistical Analysis

Variables were expressed as counts (percentages) when categorical and as mean (standard deviation, SD) or median (interquartile range, IQR) when continuous, according to data distribution. Statistical significance of the differences between groups was analyzed by Mann–Whitney test or *t* test according to the distribution of the values assessed by the D’Agostino and Pearson normality test. Correlations were assessed by Pearson or Spearman correlation analysis, as appropriate. Partial correlation analysis controlling for significant clinical variables was performed. Analyses were performed using specific software (IBM SPSS Statistic 25) and GraphPad Prism version 8.00 for Windows 11 (Redmond, WA, USA). Statistical significance was set to a *p*-value < 0.05.

## 3. Results

### 3.1. General Characteristics of the Study Population

A total of 20 patients were recruited (15 males and five females). Mean age at evaluation was 66.2 ± 12.1, mean disease duration was 4 ± 4.25 years, mean age at onset was 63 ± 13.5, mean levodopa equivalent daily dose was 375 ± 151.7 mg (calculated according to Tomlinson and Cilia [28,29]). The population presented an average BMI within the normal weight range (21.9 kg/m^2^), with a range from 17.3 to 31.6 kg/m^2^: three patients (two males, one female) were underweight, 14 patients (11 males, three females) were normal weight, and three patients (two males, one female) were overweight.

Anthropometric parameters are summarized in Table 1.

Constipation, evaluated according to the Roma III criteria, was described in 16/20 patients (80%). Dysphagia was reported only by three patients (3/20; 15%: one woman and two men), while only two presented sialorrhea (2/20; 10%, one man and one woman). To identify stool types, the Bristol Stool Scale was applied to each patient. Among the 20 patients evaluated, 10 had stool types 1 or 2 (hard stools), nine had a normal consistency (types 3 or 4) and one patient had loose or mushy stools (type 5). The risk of malnutrition was assessed using the MNA short form in 12 patients: 66.7% males (8/12) and 33.3% females (4/12). Accordingly, women showed a risk of malnutrition (score in the 8–11 range), while among men, six (75%) were at risk of malnutrition and two (25%) malnourished (score 0–7 range). Regarding sarcopenia (evaluated according to the EWGSOP2 criteria) [18], one woman was at risk of sarcopenia and four had confirmed sarcopenia. Moreover, six men were at risk of sarcopenia, four had probable sarcopenia, while five had confirmed sarcopenia. Notably, in our population, 45% of patients were diagnosed with confirmed sarcopenia. No significant correlations between sarcopenia and clinical, body composition and anthropometric parameters have been detected.

Body composition data are summarized in Table 2, while data related to sarcopenia, malnutrition, sialorrhea, dysphagia and constipation were summarized in Table 3.

Daily nutritional intake was evaluated using a food intake diary adapted to the local contest. Results were then analyzed using a Metadieta validated software version 4.7 (See Table 4).

### 3.2. Correlations Between Nutritional, Anthropometric and Nutritional Parameters with Clinical Data

**Figure 1:**
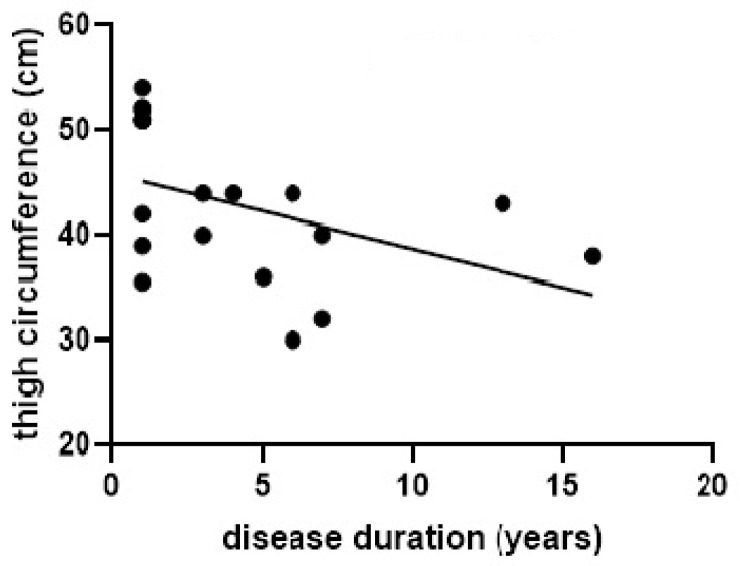
Correlation between disease duration and thigh circumference.

A significant inverse correlation was observed between disease duration and several anthropometric parameters, including BMI (*r* = −0.524, *p* = 0.02), hip circumference (*r* = −0.486, *p* = 0.03), calf circumference (*r* = −0.483, *p* = 0.03), and thigh circumference (*r* = −0.518, *p* = 0.02). After adjusting for age and sex, the inverse correlation between thigh circumference and disease duration remained significant (*r* = −0.517, *p* = 0.02) (see Figure 1).

**Figure 2:**
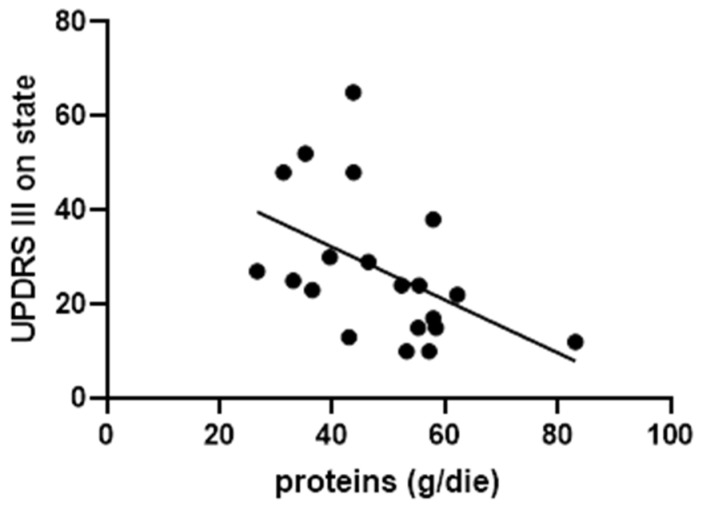
Correlation between protein intake and UPDRS III evaluated in ON phase.

As Parkinson’s disease is progressive, disease duration has a substantial impact on motor symptom severity, as reflected in the Unified Parkinson’s Disease Rating Scale Part III (UPDRS-III) score [30]. This progression makes UPDRS-III a potential confounding variable when exploring correlations between motor performance and clinical or nutritional parameters. In our cohort, a significant inverse correlation was found between daily protein intake (expressed in g/kg) and UPDRS-III scores in the ON-medication state, even after controlling for disease duration (*r* = −0.544, *p* = 0.02) (see Figure 2). This suggests that higher protein intake may be associated with better motor function, although further investigation is warranted.

Significant correlations between anthropometric parameters and disease duration are summarized in Table 5.

## 4. Discussion

This study provides a comprehensive analysis of the clinical, anthropometric, and nutritional characteristics of a cohort of patients with PD in the Volta Region of Ghana. We explored associations between nutritional status and clinical features, particularly disease duration and motor symptom severity.

Our findings confirm and expand on previous observations indicating an altered nutritional profile among PD patients in sub-Saharan Africa [8]. Notably, total daily caloric intake was lower (mean: 1542 kcal) than that reported for age-matched individuals in Italy (1933 kcal) [31]. Protein intake was also suboptimal (0.84 ± 0.18 g/kg/day), with a predominance of plant-based sources (28.9 ± 9.1 g) over animal-derived proteins (8.6 ± 7.8 g). This imbalance may impair muscle protein synthesis, particularly in older adults with anabolic resistance [32].

Patients also exhibited reduced BMI and body fat percentage, further confirming a general trend toward malnutrition, which is likely influenced by underlying socioeconomic factors [33]. Compared to our earlier findings [8], constipation prevalence in this cohort was notably higher (80% vs. 48%). This was also supported by altered stool form, as assessed by the Bristol Stool Scale. The elevated rate of constipation may be attributed to a lower daily water intake (<1 L/day), compared to the 1141 mL/day previously reported.

Dysphagia, another important contributor to malnutrition, was observed in 15% of patients. This is lower than the 39.5% prevalence reported by Gong et al. in African PD cohorts [34], possibly due to our smaller sample size, shorter disease duration, and lower levodopa equivalent daily dose (LEDD), all factors associated with dysphagia. Additionally, our data likely underestimate true dysphagia prevalence, as symptoms were self-reported during clinical evaluation. Patients may have lacked awareness of their water intake or underreported symptoms. Nonetheless, our findings underscore the importance of proactively assessing dysphagia, as it significantly increases malnutrition risk, particularly in vulnerable populations.

Sarcopenia is a debilitating condition in PD that exacerbates motor symptoms, reduces functional independence, and negatively impacts quality of life. A recent systematic review reported sarcopenia prevalence in PD ranging from 11% to 31.4% [35]. In our study, the prevalence was higher (45%), likely due to the coexisting high rate of malnutrition in this population. This may also relay on the EWGSOP2 scale used in our study. Particularly, this scale has been validated in Western populations and therefore the cut-off considered may not by properly applied to the African patients. Accordingly, our results may overestimate the problem which, however, remains an important aspect that should be considered in these patients. Further and larger studies are needed to validate the scale in specific settings.

Though considering other populations, our study is consistent with other reported cohorts in depicting malnutrition as an aggravating factor in the natural history of PD. Accordingly, Gültekin et al. evaluated the malnutrition status of a cohort of 66 Turkish PD patients using the MNA scale and identified several factors significantly associated with a higher risk of malnutrition: a reduced calf circumference, presence of dyskinesia, high Hohen and Yah stage and a daily dose of levodopa ≥ 400 mg/die [36].

Furthermore, Shidfar et al. detected a significant reduction in middle arm circumference during disease progression, as well as the calf, even though not significant [37].

We also observed a significant inverse correlation between thigh circumference and disease duration, even after adjusting for age and sex, suggesting that progressive muscle loss is closely linked to PD progression. This finding highlights a critical issue in PD: progressive muscle wasting contributes to impaired mobility, particularly in advanced disease stages, and significantly reduces independence in daily activities, as previously demonstrated by Kittipongphakorn et al. [16]. Increased patient dependency inevitably increases caregiver burden, with potential negative consequences for caregiver health and well-being [38].

All these data together highlight the importance of anthropometric measures as possible “nutritional biomarkers” associated with disease progression, further supporting the concept that nutrition assessment represents a key aspect in the clinical approach to PD patients.

Moreover, we found a significant inverse correlation between protein intake and motor impairment, as measured by the UPDRS Part III in the ON-medication state. The observed protein intake in this cohort is predominantly derived from plant-based sources, which are characterized by a lower leucine content. Accordingly, leucine, an essential branched-chain amino acid, is considered one of the most important amino acids due to its role in protein synthesis and in maintaining and preserving muscle mass [39]. Consequently, this results in a qualitatively inferior protein profile that may be directly correlated with an increased risk of sarcopenia. Although further research is warranted, this result suggests that adequate protein intake may support motor performance—either by preserving muscle mass or optimizing levodopa pharmacokinetics.

Given the insufficient intake of animal-derived proteins, our data support the hypothesis that not only the absolute quantity but also the quality and temporal distribution of protein intake significantly influence both motor performance and muscular integrity in patients with PD. These findings underscore the critical need for individualized nutritional interventions, particularly in resource-limited settings where dietary diversity may be constrained.

However, clinicians must carefully balance protein intake to avoid interference with levodopa absorption [40].

The study has several limitations. The small sample size and cross-sectional design limit generalizability and causal inference. This mainly reflects the exploratory nature of our study and is also due to the fact that the data presented here are part of the neurological assessment conducted for another study involving these patients. Therefore, the a priori sample size determination was based on that study. We are fully aware that this small sample size may have limited, on the one hand, the representativeness of patients with Parkinson’s disease in that region and, on the other hand, the robustness of the statistical analyses and consequently the interpretation of the results. Additionally, the absence of a control group represents a limitation. Including one might have helped us better understand the contribution of the disease to the development of symptoms such as sarcopenia, dysphagia, and malnutrition. However, it is worth mentioning that recruitment and data collection were further challenged by language barriers and limited access to diagnostic resources in the setting.

Nevertheless, this study involved detailed clinical and nutritional assessments and identified key markers—such as thigh circumference—that may serve as practical indicators of disease progression in PD.

## 5. Conclusions

This study confirms a high prevalence of nutritional deficiencies, sarcopenia, and altered body composition in Ghanaian patients with PD, even in the early stages of the disease. These nutritional issues are closely linked with disease duration and motor symptom severity. Our findings highlight the critical importance of early nutritional screening and intervention as part of a comprehensive, multidisciplinary approach to PD management.

As already stated, this represents an exploratory study. Although preliminary and therefore to be interpreted with caution, our results point out some interesting correlations between anthropometric parameters and disease duration in PD. Accordingly, in the future we plan to expand the cohort and include a control group to further investigate these associations, with the aim of identifying relevant clinical parameters that can be promptly addressed to achieve a more comprehensive and effective management of this chronic condition.

## Figures and Tables

**Table 1:** Anthropometric parameters of the study population.

Population	Total Cohort (Mean ± sd)	Men (Mean ± sd)	Women (Mean ± sd)	*p*
Patients	20	15	5	
Age	66.2 ± 12.12	65.6 ± 12.7	68.7 ± 11.6	0.63
Weight (kg)	58.1 ± 12.1	58.1 ± 12.2	57.7 ± 12.8	0.95
Height (cm)	162.2 ± 11.5	163 ± 11.1	161.9 ± 12.3	0.85
BMI (kg/m^2^) (range 17.3–31.6)	21.9 ± 3.4	22 ± 3.51	21.9 ± 3.58	0.95
BMR	1247.58 ± 234.9	1261 ± 232.9	1223 ± 220	0.75
Waist circumference (cm)	77.0 ± 10.3	76 ± 10.6	78 ± 11.5	0.72
Hips circumference (cm)	88.2 ± 7.4	88.2 ± 7.4	88 ± 7.98	0.95
Calf circumference (cm)	31.3 ± 3.73	31.2 ± 3.8	31.5 ± 3.79	0.88
Thigh circumference (cm)	43.0 ± 7.0	42.9 ± 7.0	43 ± 6.90	0.97

Abbreviations: BMI: body mass index; BMR: basal metabolism rate; sd: standard deviation.

**Table 2:** Body composition of the patients involved in the study.

Parameter (Abbreviation; Units)	N = 20 (Mean ± Standard Deviation)
Resistance (Rz, Ohm)	510.6 ± 63.5
Reactance (Xc, Ohm)	41.0 ± 8.4
Phase Angle (PA, °)	4.6 ± 0.8
Fat-Free Mass (FFM; kg)	47.9 ± 8.4
FFM%	83.5 ± 9.2
Fat Mass (FM; kg)	10.2 ± 7.0
FM%	16.5 ± 9.2
Total Body Water (TBW; L)	36.3 ± 7.3
Body Cell Mass (BCM, kg)	21.9 ± 5.2
Skeletal Muscle Mass (MM; kg)	27.8 ± 6.2
Appendicular Skeletal Muscle Mass Index (ASMI; kg/m^2^)	9.15 ± 0.59
ASMI (<7.4 kg/m^2^ [M]; <5.7 kg/m^2^ [F])	26.6% M; 80% F

**Table 3:** Sarcopenia, malnutrition, sialorrhea, dysphagia and constipation in our cohort.

Parameters	N	Male (=15)	Females (=5)
Dysphagia	3/20 (15%)	2	1
Sialorrhea	2/20 (10%)	1	1
Constipation	16/20 (80%)	12	4
Bristol stool chart			
Type 1–2	10/20 (50%)		
Type 3–4	9/20 (45%)		
Type 5	1/20 (5%)		
Risk of malnutrition	12	8/12	4/12
Sarcopenia (confirmed)	9/20 (45%)	5	4
Sarcopenia (at risk)	11/20 (55%)	10	1

Notes: risk of malnutrition was assessed in 12/20 patients and was reported in all of them.

**Table 4:** Nutritional data of the cohort.

	N = 20 (Mean ± Standard Deviation)
Energy (kcal/day)	1542.4 ± 284.3
kcal/kg/day	27.1 ± 4.9
Proteins (g)	48.6 ± 12.3
Lipids (g)	30.6 ± 14.0
Carbohydrates (g)	276.4 ± 65.8
Total fibres (g)	15.9 ± 5
Soluble fibres (g)	1.9 ± 0.7
Insoluble fibres (g)	10.2 ± 3.7
Animal proteins (g)	8.6 ± 7.8
Vegetal proteins (g)	28.9 ± 9.1
Saturated fatty acids (total—g)	8.2 ± 4.4
Monounsaturated fatty acids (g)	9.2 ± 7.9
Polyunsaturated fatty acids (g)	6.5 ± 2.5
Water (mL)	950 ± 350

**Table 5:** Correlations between anthropometric parameters and disease duration.

Anthropometric Parameter	Disease Duration	Adj *p* (Age and Sex)
BMI (rho, *p*)	−0.524, 0.02	Not significant
Hip circumference	−0.486, 0.03	Not significant
Calf circumference	−0.483, 0.03	Not significant
Thigh circumference	−0.518, 0.02	−0.517, 0.02

## Data Availability

The data presented in this study are available on request from the corresponding author due to ethical and legal reasons.
